# Gamma-aminobutyric acid aggravates nephrotoxicity induced by cisplatin in female rats

**DOI:** 10.15171/jrip.2016.40

**Published:** 2016-03-25

**Authors:** Elham Peysepar, Nepton Soltani, Mehdi Nematbakhsh, Fatemeh Eshraghi-Jazi, Ardeshir Talebi

**Affiliations:** ^1^Department of Physiology, Hormozgan University of Medical Sciences, Bandar Abbas, Iran; ^2^Water & Electrolytes Research Center, Isfahan University of Medical Sciences, Isfahan, Iran; ^3^Department of Physiology, Isfahan University of Medical Sciences, Isfahan, Iran; ^4^Isfahan MN Institute of Basic & Applied Sciences Research, Isfahan, Iran

**Keywords:** Gender, Gamma-aminobutyric acid, Cisplatin, Nephrotoxicity, Rats

## Abstract

**Introduction:** Cisplatin (CP) is a major antineoplastic drug for treatment of solid tumors. CP-induced nephrotoxicity may be gender-related. This is while gamma-aminobutyric acid (GABA) is an inhibitory neurotransmitter in the central nervous system that has renoprotective impacts on acute renal injury.

**Objectives:** This study was designed to investigate the protective role of GABA against CP-induced nephrotoxicity in male and female rats.

**Materials and Methods:** Sixty Wistar male and female rats were used in eight experimental groups. Both genders received GABA (50 μg/kg/day; i. p.) for 14 days and CP (2.5 mg/kg/day; i. p.) was added from day 8 to the end of the study, and they were compared with the control groups. At the end of the study, all animals were sacrificed and the serum levels of blood urea nitrogen (BUN), creatinine (Cr), nitrite, malondialdehyde (MDA), and magnesium (Mg) were measured. The kidney tissue damage was also determined via staining.

**Results:** CP significantly increased the serum levels of Cr and BUN, kidney weight, and kidney tissue damage score in both genders (*P*<0.05). GABA did not attenuate these markers in males; even these biomarkers were intensified in females. Serum level of Mg, and testis and uterus weights did not alter in the groups. However, the groups were significantly different in terms of nitrite and MDA levels.

**Conclusion:** It seems that GABA did not improve nephrotoxicity induced by CP-treated rats, and it exacerbated renal damage in female rats.

**Implication for health policy/practice/research/medical education:**

In an experimental study, we found, gamma-aminobutyric acid did not improve nephrotoxicity induced by CP-treated rats, and it exacerbated renal damage in female rats.

## Introduction

Cisplatin (CP) (cis-diamminedichloroplatinum II) is a major antineoplastic drug for treatment of solid tumors (1). One of the side effects of CP is nephrotoxicity which is specified by decreasing in glomerular filtration rate and renal blood flow (2), increaseing serum levels of blood urea nitrogen (BUN) and creatinine (Cr), and enhancing kidney weight (KW) and histopathological changes (3,4). Inflammation, oxidative stress, and apoptosis participate in nephrotoxicity (5,6). On the other hand sex-related differences have been reported in CP-induced nephrotoxicity in animal models (7,8). This is while clinically men with chronic renal disease exhibit more rapid reduction in kidney function (9). In addition, the two genders show various responses to administration of supplements against CP-induced nephrotoxicity (3,4,10). Gamma-aminobutyric acid (GABA) is well known as an inhibitory transmitter in the central nervous system of vertebrates (11). Also, there are remarkable quantities of GABA in extra-neural tissues such as kidney (12). GABA has dilatory and vasodilatory effects on blood vessels (13,14). GABA induces regenerative and immunoinhibitory effects of β-cell in diabetes type 1 (15) and protects kidney against injury induced by renal ischemia reperfusion (16).

## Objectives

This present study was designed to investigate the protective role of GABA against CP-induced nephrotoxicity in male and female rats.

## Materials and Methods

### Animals

This research was performed on 30 adult male (245.71±3.91 g) and 30 adult female (185.36±1.91 g) Wistar rats (Animal Centre, Isfahan University of Medical Sciences, Isfahan, Iran). The animals were housed under standard conditions with 12-hour light/12-hour dark cycle with free access to water and rat chow. The research protocols were in advance approved by the Isfahan University of Medical Sciences Ethics Committee.

### Drugs

CP and GABA were purchased from Mylan S.A.S. (France) and Sigma (St. Louis, MO, USA), respectively.

### Experimental protocol

Wistar rats were randomly assigned to eight groups, and were treated for 14 days.

Group 1 (male, n = 6), named as vehicle, received intraperitoneal (i.p.) saline during the study.

Group 2 (male, n = 6), named as GABA, received GABA (50 μg/kg/day; i. p.) for 14 days and saline was added from day 8 until the end of the study.

Group 3 (male, n = 8), named as CP, received saline (i.p.) for 14 days and CP (2.5 mg/kg/day; i. p.) was added from day 8 until the end of the study.

Group 4 (male, n = 8), named as GABA+CP, received GABA (50 μg/kg/day; i. p.) for 14 days and CP (2.5 mg/kg/day; i. p.) was added from day 8 until the end of the study.

Groups 5 (n = 6), 6 (n =6), 7 (n = 8), and 8 (n = 8) were female rats that received regimens the same as groups 1-4, respectively.

The animals’ bodyweight was recorded daily. At the end of the study (day 15), all animals were anesthetized to obtain blood samples via heart puncture. Then, the rats were sacrificed and the kidneys, uterus, and testis were removed and weighed rapidly. The left kidney was subjected to histopathological investigations, and the right kidney was homogenized and centrifuged. The supernatant and the removed serum samples were kept at -20^o^C until measurement.

### Measurements

Cr, BUN serum levels were measured with quantitative kits (Pars Azmoon) using an automatic analyzer (Technicon, model RA1000). Magnesium (Mg) level was determined using quantitative kits (Pars Azmoon, Iran) by a spectrophotometer. The serum and renal levels of nitrite (nitric oxide metabolite) were measured using a colorimetric assay kit (Promega Corporation, USA). The serum level of malondialdehyde (MDA) was quantified according to the manual methodology. At first, a solution was prepared including 15 g trichloroacetic acid, 0.375 g thiobarbituric acid, and 2 ml hydrochloric acid in total volume of 100 cc. Then, 2 cc of the prepared solution and 1 cc of the sample were mixed. The mixture was incubated in boiling water bath at the temperature of 100°C for 60 minutes and after cooling; the mixture was centrifuged at 1000 g for 10 minutes. Finally, the absorbance was measured at 535 nm.

### Histopathological procedures

The kidney tissues were fixed in 10% formalin solution and embedded in paraffin for hematoxylin and eosin histopathological staining. Kidney tissue damage score (KTDS) was determined in the range of 0-4 by a pathologist.

### Ethical issues

Prior to the experiment, the protocols were confirmed to be in accordance with the Guidelines of Animal Ethics Committee of Isfahan University of Medical Sciences. The experimental procedures were approved in advance by the Isfahan University of Medical Sciences Ethics Committee.

### Statistical analysis

Data were presented as mean ± SEM. The groups were compared with regard to the levels of BUN, Cr, MDA, Mg, nitrite, KW and bodyweight change (∆BW) by analysis of variance (ANOVA) analysis followed by LSD as the post hoc. The Kruskal-Wallis and Mann-Whitney U tests were employed to compare the KTDS. *P* values <0.05 were considered statistically significant.

## Results

### Effect of CP and GABA on serum levels of BUN, Cr, Mg, nitrite and MDA, and kidney level of nitrite and KTDS

**Figure 1:**
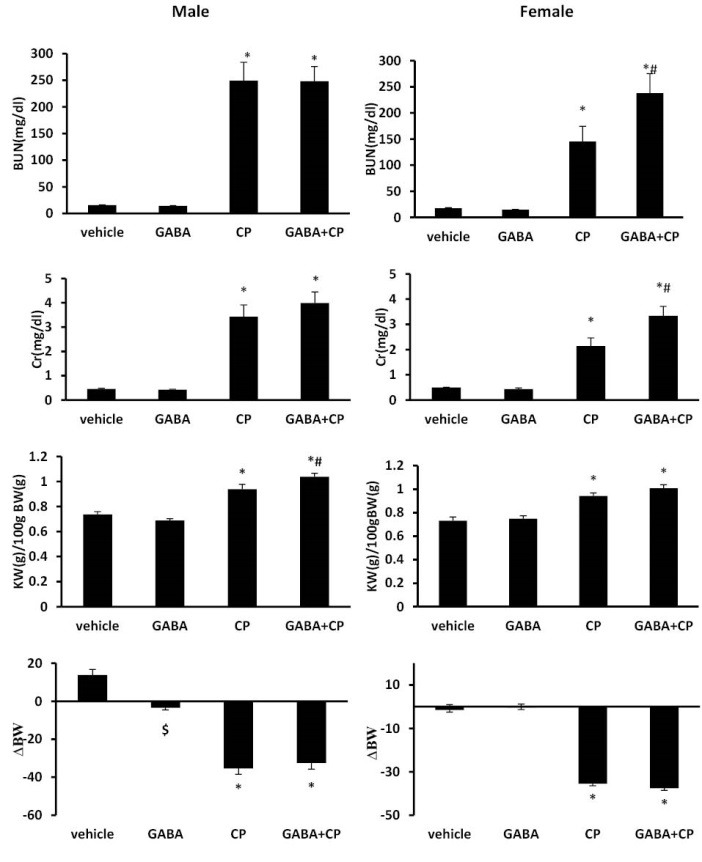
Serum levels of blood urea nitrogen (BUN) and creatinine (Cr), bodyweight change (∆BW) and kidney weight (KW) g/100 g BW in experimental groups. The data were reported as mean ± SEM. The signs described significant differences (*) from vehicle and GABA groups; (#) from CP group; and ($) from vehicle group (*P*<0.05). GABA and CP stand for gamma-aminobutyric acid and cisplatin, respectively.

CP alone significantly increased the serum levels of Cr and BUN, and KTDS in male and female rats, when compared with the vehicle and GABA groups (*P*<0.05). Administration of GABA aggravated the increasing levels of BUN, Cr, and KTDS induced by CP in comparison with the CP group in female gender (*P*<0.05), while such observation was not seen in males (Figure 1, Table 1). No significant difference was observed in serum level of Mg between the groups. Kidney nitrite level significantly decreased in the CP and GABA+CP groups in both genders (*P*<0.05). In addition, GABA alone increased kidney nitrite level only in females (*P*<0.05). Serum nitrite level enhanced in female rats treated by CP alone, but administration of GABA reduced this alteration (*P*<0.05). This finding was not seen in male groups (Table 2). CP elevated serum level of MDA in female rats (*P*<0.05); however, GABA could not reverse this result. The combination of GABA and CP induced significant increase in serum level of MDA in males (*P*<0.05; Table 2).

**Table 1:** The data for score of the kidney tissue. The numbers in the table are represented the numbers of kidneys in each grade score

	**Group**	** KTDS**					**N**
**Gender**	**Treatment / Score**	**0**	**1**	**2**	**3**	**4**	
Male	Vehicle	4	2				6
	GABA	5	1				6
	CP*			3	5		8
	GABA+CP*			2	6		8
Female	Vehicle	3	3				6
	GABA	2	4				6
	CP*			7	3		8
	GABA+CP*#			2	6		8

Abbreviations: KTDS, Kidney tissue damage score; GABA, gamma-aminobutyric acid; Cp, cisplatin. Grading scale is as follows: 0 = indistinguishable from vehicle; 1 = minimal, ≤ 25% cortex affected; 2 = mild, >25% and ≤50% cortex affected; 3 = moderate, > 50% and ≤ 75% cortex affected; 4 = severe, > 75% cortex affected. The signs described significant differences (*) from vehicle and GABA groups; (#) from CP group (*P*<0.05).

**Table 2:** Serum and kidney levels nitrite, serum levels of malondialdehyde (MDA) and magnesium (Mg), testis and uterus weight g/ 100 g BW in experimental groups

**Group**		**Serum nitrite (µmol/L)**	**Kidney nitrite (µmol/g tissue)**	**Serum MDA (µmol/L)**	**Serum Mg (mg/dL)**	**Testis weight (g/100 g BW)**	**Uterus weight (g/100 g BW)**
**Gender**	**Treatment**						
Male	Vehicle	16.95 ± 2.81	0.28 ± 0.02	4.77 ± 0.47	2.17 ± 0.03	0.99 ± 0.03	-
	GABA	15.38 ± 2.66	0.29 ± 0.01	5.14 ± 0.24	2.13 ± 0.01	1.10 ± 0.03	-
	CP	46.65 ± 20.31	0.17 ± 0.03*	6.57 ± 0.18	2.21 ± 0.01	1.07 ± 0.03	-
	GABA+CP	56.40 ± 32.84	0.18 ± 0.03*	8.96 ± 2.12*	2.23 ± 0.07	1.10 ± 0.04	-
	P value	0.57	0.01	0.06	0.35	0.22	-
Female	Vehicle	17.25 ± 5.84	0.20 ± 0.01	5.97 ± 0.66	2.19 ± 0.03	-	0.05 ± 0.004
	GABA	13.98 ± 3.95	0.30 ± 0.02^$^	4.84 ± 0.25^$^	2.09 ± 0.11	-	0.05 ± 0.006
	CP	45.90 ± 12.92*	0.19 ± 0.03^&^	5.99 ± 0.24^&^	2.17 ± 0.06	-	0.04 ± 0.004
	GABA+CP	16.87 ± 4.52^#^	0.15 ± 0.01^&^	6.17 ± 0.30^&^	2.14 ± 0.07	-	0.04 ± 0.004
	P value	0.02	0.008	0.06	0.84	-	0.47

The data are reported as mean ± SEM. The signs indicate significant differences (*) from vehicle and GABA groups; (#) from CP group; ($) from vehicle group; and (&) from GABA group (P< 0.05). GABA and CP stand for gamma-aminobutyric acid and cisplatin, respectively.

**Figure 2:**
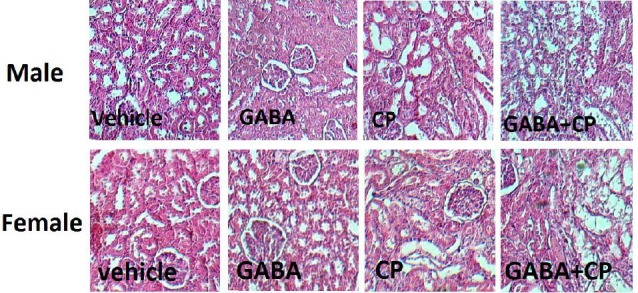
The images of kidney tissue (magnification × 100) in experimental groups. GABA and CP stand for gamma-aminobutyric acid and CP, respectively.

### Effect of CP and GABA on BW, KW, UW and TW

Administration of GABA alone decreased BW when compared with the vehicle group in male gender (*P*< 0.05), and such result was not seen in female rats. This is while administration of CP induced significant BW loss in both genders, (*P*<0.05) and the combination of GABA and CP did not improve this parameter in both genders (Figure 1).

CP alone increased KW (g/100 g BW) in both male and female rats (*P*<0.05). Co-administration of GABA and CP did not change KW when compared with the CP alone-treated group in females, but this enhancement was aggravated in males (*P*<0.05; Figure 1).

Significant changes in UW (g/100 g BW) and TW (g/100 g BW) were not observed (Table 2).

The images of kidney tissue from all experimental groups were shown in Figure 2.

## Discussion

In the present study, we attempted to investigate the effect of GABA on CP-induced nephro­toxicity in male and female rats. The major finding indicated that GABA did not have nephroprotective effect against CP-induced nephrotoxicity in both genders; even it aggravated renal damage in female rats.

Our results showed that administration of CP alone in male and female rats induced an increase in the levels of kidney func­tion markers including BUN and Cr, which was confirmed by pathology data and KW changes. These findings are in agreement with those reported in other studies (7,8). CP induces nephrotoxicity via oxidative stress, inflammation, and cell necrosis (5,6). In this study, GABA administration had no ameliorating effect on nephrotoxicity induced by CP in male rats. In contrast, oral administration of GABA mitigated renal dysfunction induced by CP (17). Possibly, the difference in results is due to different protocols of studies. We used continues dose of CP, whereas Ali et al (17) applied single dose of CP. Our pervious findings confirmed this hypothesis (4,18). Results of this study showed that administration of GABA could not ameliorate nephrotoxicity induced by CP in female; even it exacerbated renal damage characterized by more increment in BUN and Cr levels and KTDS. Accordingly, several studies indicated that various compounds such as erythropoietin (10), L-arginine (3), losartan (4), and vitamin E (19) lead to a greater damage in females treated by CP. This is probably related to interaction between these agents and sex hormones or CP. It is documented that estrogen itself increases renal damage induced by CP in ovariectomized rats (20), even it affects the positive role of erythropoietin (21), vitamin E, vitamin C, and losartan (22). CP administration reduced BW. This finding, which is in agreement with other report (7), is probably due to gastrointestinal disorders such as diarrhea. Moreover, BW loss induced by CP was not ameliorated after administration of GABA.

Nitric oxide level is a key regulator of cardiovascular homeostasis. It was observed that CP alone differently affected renal and serum levels of nitrite. There are some reports on the effects of CP and gender on nitrite level (3,4,7,8,10,19,23,24) while the involved mechanisms are not well known. It seems that CP elevated serum level of inducible NOS and reduced endothelial NOS in kidney. Administration of GABA reduced increment of nitrite serum level induced by CP in female; probably via reducing inducible NOS. Either administration of CP or GABA + CP decreased kidney nitrite level in both genders; possibly through reducing inducible NOS, whereas GABA alone stimulated an increase in kidney nitrite level. It is reported that GABA, as a component of the aqueous extract of red rice fermented with *Monascus ruber*, produces nitric oxide through stimulating vascular endothelial cells (25).

CP administration increased serum MDA level in both genders and GABA could not attenuate it in female rats. CP induced lipid peroxidation by elevating MDA (18). We observed that GABA intensified increased serum MDA level in males. Despite GABA had no effect on other parameters such as BUN and Cr, it seems that it aggravated lipid peroxidation in male rats.

## Conclusion

We concluded that GABA as an antioxidant or as a candidate for reduction of serum glucose level did not improve nephrotoxicity induced by CP in male and female rats. The greater nephrotoxicity induced by GABA and CP in female rats suggested to test GABA in a model of diabetic rats treated with CP.

## Authors’ contribution

EP and FEJ were involved in experimental procedure and data analysis. NS and MN designed the study, verified data analysis and wrote the article. AT was involved in study design and pathology procedure and analysis. MN, edited the final manuscript. All authors read and signed the final paper.

## Conflicts of interest

The authors have no conflict of interests to declare.

## Ethical considerations

Ethical issues (including plagiarism, data fabrication, double publication) have been completely observed by the authors.

## Funding/ Support

This research was supported by Isfahan University of Medical Sciences.
